# The Use of PGPB to Promote Plant Hydroponic Growth

**DOI:** 10.3390/plants11202783

**Published:** 2022-10-20

**Authors:** Ashley A. Stegelmeier, Danielle M. Rose, Benjamin R. Joris, Bernard R. Glick

**Affiliations:** 1 Ceragen Inc., 151 Charles St W, Suite 199, Kitchener, ON N2G 1H6, Canada; 2 Department of Biology, University of Waterloo, 200 University Avenue West, Waterloo, ON N2L 3G1, Canada

**Keywords:** hydroponics, plant growth-promoting bacteria, plant stress, plant growth, ACC deaminase, siderophores, food production, cannabis, space

## Abstract

Improvements to the world’s food supply chain are needed to ensure sufficient food is produced to meet increasing population demands. Growing food in soilless hydroponic systems constitutes a promising strategy, as this method utilizes significantly less water than conventional agriculture, can be situated in urban areas, and can be stacked vertically to increase yields per acre. However, further research is needed to optimize crop yields in these systems. One method to increase hydroponic plant yields involves adding plant growth-promoting bacteria (PGPB) into these systems. PGPB are organisms that can significantly increase crop yields via a wide range of mechanisms, including stress reduction, increases in nutrient uptake, plant hormone modulation, and biocontrol. The aim of this review is to provide critical information for researchers on the current state of the use of PGPB in hydroponics so that meaningful advances can be made. An overview of the history and types of hydroponic systems is provided, followed by an overview of known PGPB mechanisms. Finally, examples of PGPB research that has been conducted in hydroponic systems are described. Amalgamating the current state of knowledge should ensure that future experiments can be designed to effectively transition results from the lab to the farm/producer, and the consumer.

## 1. Introduction

Between 1900 and 2022, the human population has quadrupled to approximately 8 billion people, with current projections expecting 10 billion inhabitants by 2050. Our existing food production systems are insufficient to meet this population increase, as it is estimated that over 800 million people already suffer from hunger worldwide, while 3.5 billion suffer from deficiencies of at least one essential nutrient [1]. As income growth continues in low- and middle-income countries, there will be increasing demand on the food production system, and this will be in addition to the food required to meet the needs of 2 billion additional people [2]. Thus, it is estimated that to meet global food requirements, agricultural production needs to increase by at least 0.5% each year by 2050 [3].

The first major problem in respect to increasing food production is that ~50% of all the world’s habitable land is already used for agriculture [4]. Of the remaining portion, 37% is forests, 11% is shrubs and grasslands, 1% is freshwater coverage, and 1% is urban areas. Historically, one of the major ways that humans have increased food production is by expanding the amount of agricultural land. One thousand years ago agricultural land made up only 4% of all habitable land, in comparison to 50% today [5]. Without an increase in productivity this would mean that 50–100% of the remaining habitable land would need to be converted into agricultural land to feed the human population, an idea that is completely unsustainable from a climate change or ecological standpoint.

The second major problem is that the quality of current agricultural land quality is on the decline. Over 50% of current agricultural land is moderately or severely impacted by soil degradation processes including erosion, acidification, compaction, salinization, and contamination [1]. Soil erosion is considered the largest contributing factor to soil degradation, accounting for a loss of 75 billion tons of fertile soil each year [6]. This means that each year, without agricultural expansion and its associated environmental costs, there is less available or productive land to produce the food our population requires.

The third major problem is that, due to climate change, the productivity of our current crops is on the decline. It is predicted that rising global temperatures and erratic weather conditions will lead to major decreases in staple crop production including maize (20–45%), wheat (5–50%), and rice (20–30%) [1]. Additionally, up to 30% of annual agricultural production could be lost due to increasing phytopathogen and pest pressure as temperatures continue to rise [7].

The fourth major problem is freshwater availability. Globally over 50% of the world relies on groundwater for their daily needs, and over 35% of agricultural irrigation uses groundwater as its source [8]. Unsustainable agricultural use combined with droughts has led to a decline in groundwater in many geographic areas [8]. In 2010 it was estimated that over 80% of people live in areas that have a high likelihood of water security threats [9]. As such, reducing agricultural usage is essential to ensure adequate fresh water supplies to meet the demands of the growing human population.

While overcoming these food production challenges may seem like an impossible task, controlled environment agriculture (CEA) systems are proving a promising solution. CEA is the process of growing crops in a manner that gives the farmer partial to full control over the environmental variables that affect plant growth. Low tech systems such as row covers, low tunnels, high tunnels, and net covers are often referred to as “protected agriculture” [10]. These systems provide less control over environmental systems in comparison to their “high tech” counterparts. High tech-controlled environment systems include greenhouses and indoor farms [11].

While the earliest recorded cases of greenhouse use stretch back to 14 CE during the Roman period, commercial-scale use did not begin until the 20th century in the Netherlands [10]. During World War II the Netherlands’ greenhouses sustained extensive damage, and during reconstruction after the war engineers in the Venlo region developed the tall, glass, multi-span greenhouse that is often seen today. Significant efforts were made post war to ensure food security in the Netherlands, including the construction of many Venlo style greenhouses for vegetable production [12]. Since the initial introduction of commercial CEA production in the Netherlands, greenhouse use has expanded across the globe [13]. Significant improvements in glazing materials, lighting sources, and growing systems have greatly increased the yield potential of greenhouse production in comparison to field agriculture. One significant advancement was the introduction of hydroponic crop production.

Hydroponics is the method of growing plants in soilless systems where the nutrients for growth are provided via a water based nutrient solution. In comparison to soil-based systems hydroponic systems offer significantly higher yields (~14× higher kg/fresh weight/m^2^ in vertically farmed lettuce) [14] and faster growth times as nutrients are more readily available to the plant and root growth is not hindered by mechanical interference from the soil [15,16]. Hydroponic and aquaponic systems can also be installed in areas where the soil or climate is unsuitable for traditional agricultural and aquaculture production [17]. This can help increase food production in areas where the soil has become contaminated, acidified, and/or salinized. Additionally, when combined with a greenhouse structure, hydroponic systems offer year-round local production of fresh fruits and vegetables in colder climates [18]. Furthermore, hydroponic systems have been shown to use less fertilizer than soil-based production and up to 90% less water [10,19].

While the advantages of hydroponics offer significant promise with regard to feeding the growing global population, there are still hurdles that must be overcome for this technology to overtake conventional soil-based farming. First, greenhouses have a high initial setup cost due to the materials required (namely steel and glass or plastic depending on the glazing selected) [20]. Second, hydroponic systems are far more complicated to operate than conventional growing equipment given the number of environmental variables under the growers’ control. Third, the margin for error is much lower and system failures or electrical outages can cause serious impacts on plant health [21]. Finally, pathogens can easily spread throughout an entire crop due to the proximity of plants and recirculated nutrient solution [22].

While hydroponic greenhouse growers have been able to optimize most environmental factors such as lighting, CO_2_, heating, and fertilizers, to date the plant microbiome has largely been ignored. Plant growth promoting bacteria (PGPB) offer significant benefits to plants in soil-based systems [23]. This includes helping the plant uptake nutrients, promoting growth, stress regulation, and pathogen prevention. However, many plant growth-promoting microbes found in soil cannot make the transition to hydroponic environments [24]. Nevertheless, PGPB offers a unique solution to several of the largest problems in hydroponic production by preventing pathogen outbreaks, improving the plants’ response to environmental stress, and increasing crop yield per m^2^ all of which reduces the payback period of the initial capital investment.

In this review, the types of hydroponic systems currently in use, the beneficial traits of plant growth promoting bacteria, the current research to date on the hydroponic plant microbiome, and the current research to date on the use of PGPB in hydroponic systems are discussed.

## 2. Hydroponic Systems

The earliest known records of plants grown in a hydroponic like system come from descriptions of the Hanging Gardens of Babylon built along the Euphrates River circa 600 BCE, in what is now Iraq. However, widespread commercial use of these systems did not begin until the late 1900’s when hydroponic growing systems gained popularity with greenhouse vegetable growers in Europe and North America [10]. In the 1700’s researchers began experimenting with growing plants in what would become known throughout the research community as “water culture”. In water culture studies researchers had the ability to control specifically what nutrients were available to the plants through the addition of chemicals to the water in the system. As such, this technique was used in many of the studies that determined the nutritional requirements of plants [25].

**Figure 1:**
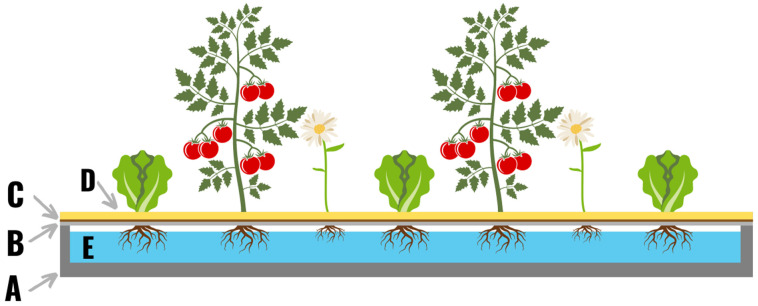
The original hydroponic system built by Gericke in 1929. A roll of bituminous roofing paper (36 ft × 3 ft) was folded up on every edge 6 inches to form a trough (**A**). On top of the trough a layer of wire netting was attached (**B**), and a layer of burlap (**C**) was added on top of the wire. A one-inch layer of sand (**D**) was added on top as a substrate for the plants to grow in. The trough was filled with nutrient solution (**E**), and a variety of crops were planted in the system.

Throughout the 19th and early 20th century water culture was used solely for the purposes of academic research. However, this changed in 1929 when Dr. William Frederick Gericke from the University of California published a paper outlining the potential for growing commercial crops in water culture [26]. In Gericke’s study, a water reservoir was created using bituminous roofing paper, topped with wire mesh, burlap, and sand (which was used as a growing media for the plants; Figure 1). The reservoir was then filled with water supplemented with “the elements required for growth of plants in water” [26]. Gericke used this system to grow numerous types of plants, including those intended for commercial food production. Gericke states in his 1929 paper that the “results obtained warrant serious consideration of this method for production of certain crops grown on an intensive scale”, and the field of hydroponic crop production was born.

While most modern hydroponic systems have come a long way from that reservoir made with roofing paper, the core principles of the technique remain the same. In 1938 Gericke defined hydroponics as the “art and science of crop production in liquid culture media” [25]. The term hydroponics was suggested by Dr. William Setchell from the University of California to separate the previous research focused water culture technique and the new commercial focused growing technique [27]. The term itself is a combination of the words, “hydro” (meaning water) and “ponos” (meaning labor) [25,27].

Most modern hydroponic systems can be categorized into seven main types. These systems are primarily differentiated by how the nutrient solution is applied to the root system of the plant.

### 2.1. Wicking Systems

**Figure 2:**
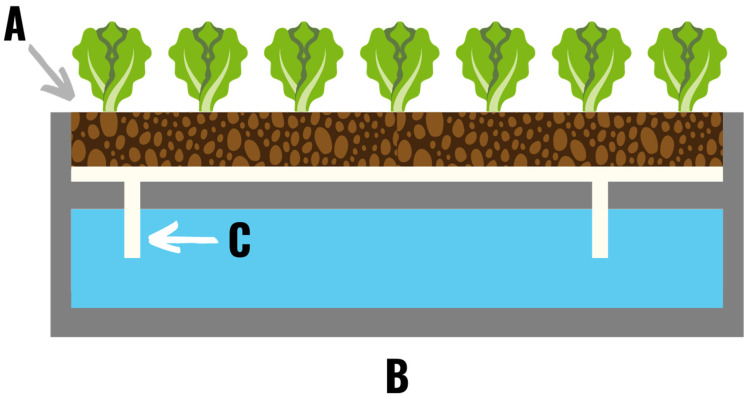
A wicking hydroponic system in which plants are grown in an absorbent soilless grow media (**A**). Nutrient solution is transferred from the reservoir (**B**) to the plant roots via an absorbent wicking material (**C**).

A wicking system consists of a grow tray filled with an absorbent soilless grow media, a storage tank for the nutrient solution, and an absorbent wick that transfers nutrient solution from the tank to the grow media (Figure 2). This system is primarily used for small plants such as leafy greens and herbs, and is typically not used for commercial applications or larger fruiting plants such as tomatoes or cucumbers [28]. Major advantages for this type of system are that it is easy to construct, does not require the use of a pump, and can be used for small setups that are suitable for the home or office [29]. Semananda and colleagues determined that a wicking system containing tomatoes reduced nutrient leachate and performed equivalent or better than surface irrigation [29].

### 2.2. Ebb and Flow (Flood and Drain)

**Figure 3:**
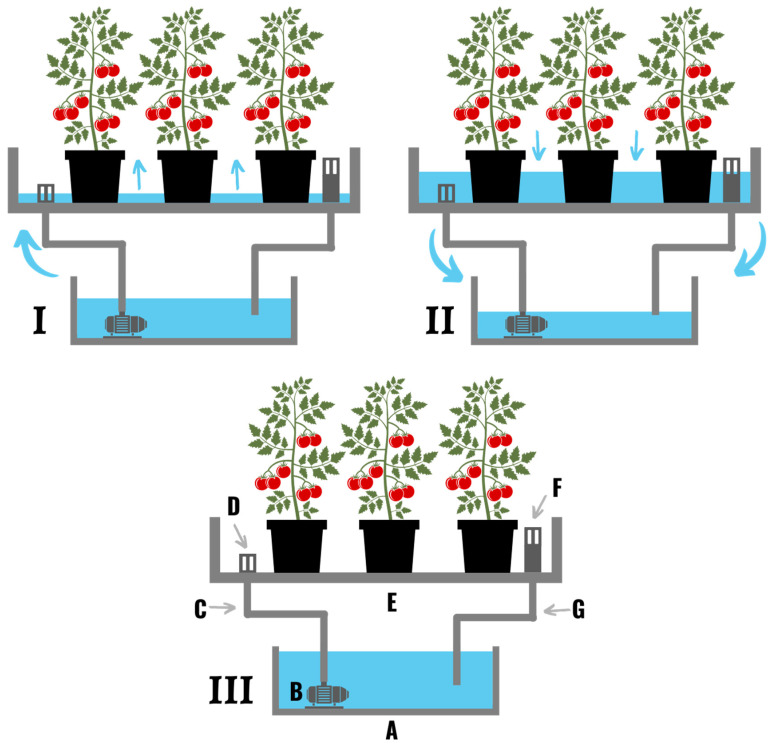
An ebb and flow or flood and drain hydroponic system in which nutrient solution is pumped from an external reservoir (**A**) over the plants at set intervals during the day. (**I**) During the fill stage a timer turns on the pump (**B**), which pumps the nutrient solution up through a pipe or tube (**C**), through the fill inlet/drain (**D**), and into the flood tray (**E**). An overflow drainpipe (**F**) sits several inches above the fill inlet/drain, and prevents the system from overflowing. (**II**) If the tray starts to overflow nutrient solution will return to the reservoir (**A**) through the tube connected to the overflow drainpipe (**G**). Once the pump turns off, the nutrient solution drains back down the fill tube (**C**), through the pump (**B**), and into the reservoir (**G**). (**III**) After the pump turns off the flood table (**E**) drains completely back into the reservoir (**A**) and sits empty until the next pump cycle.

In an ebb and flow hydroponic system plants are grown in a tray that is regularly flooded with nutrient solution at set times throughout the day (Figure 3). Nutrient solution is pumped from a reservoir into the grow tray, where the liquid is kept at a specific level (by means of an overflow drain) for a set amount of time before the pump shuts off, allowing the nutrient solution to drain back down the input pipe (Daud, 2018; [16,30]. Using this system, plants are typically grown either in pots filled with soilless media (for larger plants), or in grow plugs housed in trays (for seedlings). Ebb and flow systems are primarily used for seedling cultivation in commercial settings. Kale and cherry tomatoes yields from ebb and flow systems had larger gross returns than basil and chipotle peppers [31]. Compared to top sprinkle irrigation, ebb and flow systems improved tomato root parameters and stem diameter by 9–45% [32].

### 2.3. Drip Irrigation

**Figure 4:**
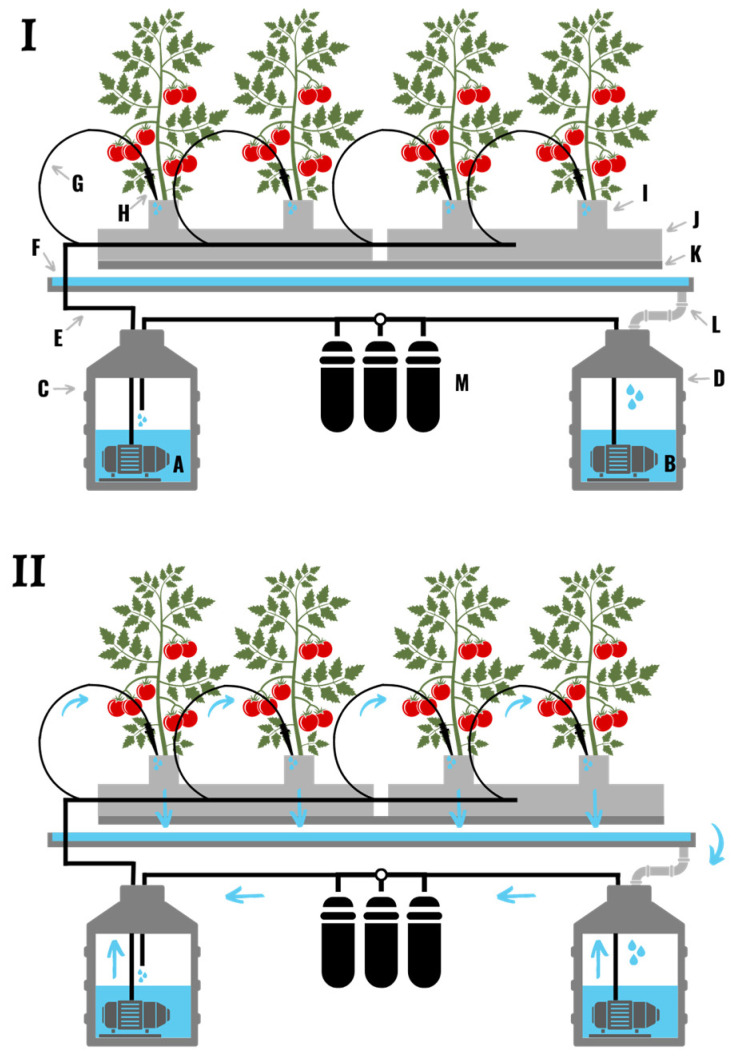
(**I**) A drip irrigation system comprised of water pumps (**A**,**B**), two reservoirs (**C**,**D**), a watering line/pipe (**E**), a drain trough (**F**), drip lines (**G**), drip stakes (**H**), a smaller grow media cube than the transplanted plant was propagated in (**I**), a large grow media slab (**J**), a platform (**K**), a drainpipe (**L**), and a water purification system (**M**). (**II**) Several times a day water is pumped from the storage reservoir, through the drip lines and over the roots of the plants. The nutrient solution that runs off the roots of the plant is usually filtered and sterilized to eliminate any pathogens prior to recirculation. The nutrients content of the collected nutrient solution is then adjusted back to desired levels before it is recirculated back onto the plants. In non-recirculated systems water is collected in a drainage trough and fed back to a central reservoir. The nutrient solution is then filtered and sterilized to remove particulates and pathogens.

Drip irrigation systems are the primary hydroponic system used in commercial production for larger fruiting crops such as tomatoes, cucumbers, peppers, and strawberries (Figure 4). In this type of system plants are typically grown on an inert substrate such as rockwool or coco coir. Nutrient solution is stored in a separate tank and pumped over the roots of the plants several times a day via drip lines at an interval that keeps the substrate moist. These drip lines are typically composed of a thin, black, polyethylene tube attached to a drip spike that is inserted into the growing media [19]. Drip irrigation has been shown to improve tomato nutritional value and antioxidant levels compared to furrow irrigation [33].

Drip systems can either be recirculating or non-recirculating (also known as recovery or non-recovery). In recirculating systems nutrient solution is collected and reused to reduce water and fertilizer use [34]. Cabernet Sauvignon grapevines had higher photosynthesis levels and healthier roots in recirculating systems compared to aerated solution culture [34]. In commercial non-recirculating systems collected nutrient solution is applied to the roots of the plants, and runoff is not collected or reused. This type of system has larger water and fertilizer requirements than that of a recirculating system [35]. Engineers have been creating inexpensive small scale recirculating drip technology to reduce runoff and decrease the need for non-recirculating systems [35].

### 2.4. Nutrient Film Technique (NFT)

The nutrient film technique was invented by Allen Cooper in 1965 [36]. In Cooper’s NFT system “a very shallow stream of water containing all the dissolved nutrients required for growth is recirculated past the bare roots of crop plants in a water-tight gully” [37]. The original NFT system built by Cooper consisted of concrete ditches lined with polyethylene film. These ditches were on a 1^o^ slope to allow the nutrient solution to run “downhill” past the plant roots. These concrete ditches were replaced by extruded polyethylene hydro channels in the late 1970’s, which were replaced by rigid white PVC channels in the 1980’s [19].

**Figure 5:**
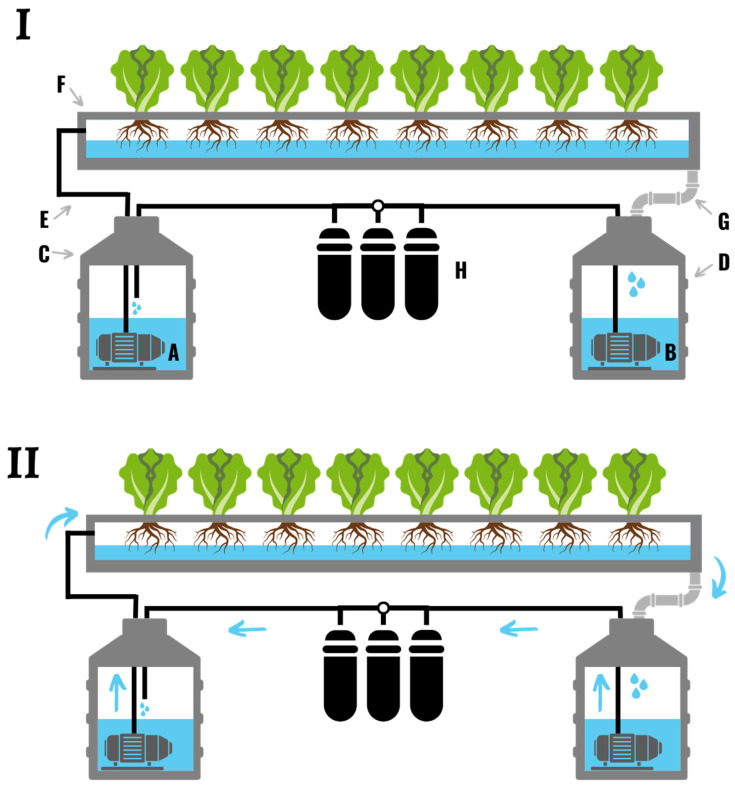
(**I**) A nutrient film hydroponic system comprised of two water pumps (**A**,**B**), two reservoirs (**C**,**D**), a watering line (**E**), a grow trough (**F**), a drainpipe (**G**), and a sterilization system (**H**). (**II**) Water is pumped from the storage reservoir, through the watering line, and down the grow trough where it runs around the roots of the plant. The nutrient solution then drains out of the grow trough, and is fed back to a central reservoir through the drainpipe. The nutrient solution is then filtered and sterilized to remove particulates and pathogens.

**Figure 6:**
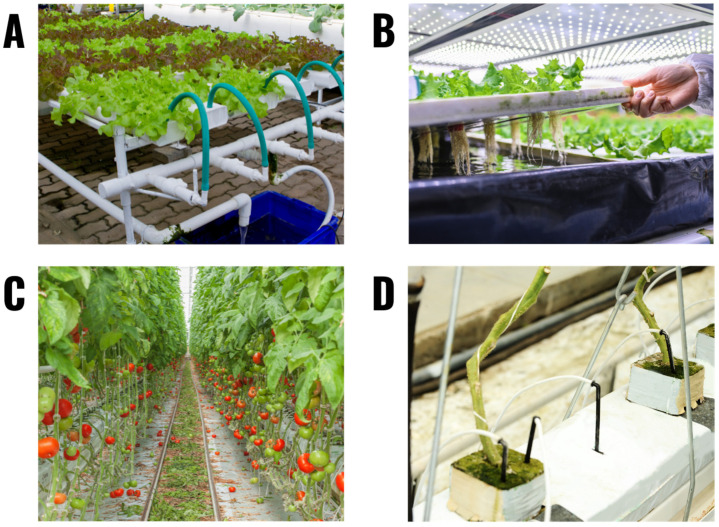
Examples of commercial or research hydroponic systems. (**A**) A small NFT system growing lettuce. (**B**) An indoor deep water culture system growing lettuce. (**C**) A tomato greenhouse using drip irrigation. (**D**) A close up of the drip irrigation system used in the tomato greenhouse. The tomato plants in this system are growing in rockwool cubes placed upon rockwool slabs.

In modern NFT systems water is pumped from a water reservoir and into a slanted PVC channel where it runs past the roots of the plants as it travels “downhill”. The nutrient solution is then collected, and in commercial systems filtered (to remove any debris) and sterilized prior to recirculation (Figure 5 and Figure 6). In commercial settings NFT systems are primarily used for smaller, leafy crops such as lettuce [19]. Typically, lettuce plants are germinated in one-inch cubes of inert growing media such as rockwool, then transferred to the NFT system after the first few leaves have appeared. The typical spacing for full lettuce heads in NFT systems is eight inches between the centers of each head. Lettuce plants harvested at the “baby leaf” stage may be grown closer together than the standard eight-inch centers. A lettuce hydroponic study concluded that lettuce grown in NFT systems were 6–10% larger than Deep Film Technique (a variation of NFT in which the water level in the channel is kept in the range of several centimeters deep in comparison to the thin film used in NFT) and floating systems [38].

### 2.5. Deep Water Culture

**Figure 7:**
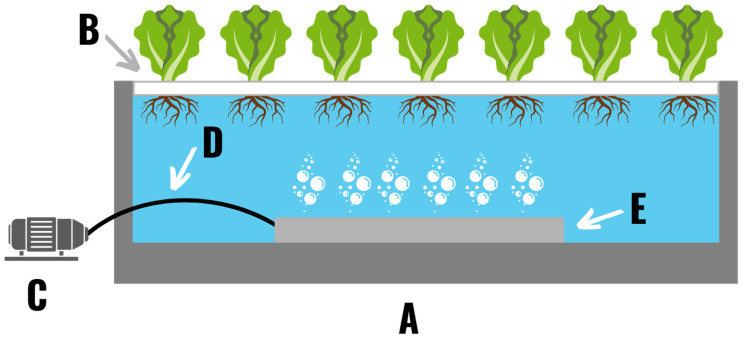
A deep-water culture hydroponic system comprised of a reservoir (**A**), a floating raft (**B**), an air pump (**C**), an airline (**D**), and a bubbler (**E**). Plants are placed in the grow raft and suspended in the nutrient solution. Proper oxygenation of the root zone is ensured by bubbling air through the nutrient solution.

In deep water culture systems plants are grown in a reservoir in which the nutrient solution is aerated with a form of mechanical aeration (often in the form of an air stone and pump). Part of the plant is supported above the nutrient solution by a floating or suspended platform so that just the roots are submerged in the nutrient solution (Figure 7). Floating rafts or plates are typically constructed out of polystyrene with holes drilled through the raft for each plant. In commercial settings deep water culture systems are primarily used for small leafy plants such as lettuce or herbs [19]. Outside of research use, deep water culture is sometimes divided into two sub-variants based on reservoir depth. The term “deep water” culture is typically used to refer to reservoirs with depths greater than 10 inches, while “shallow water” culture systems typically have a depth of less than 10 inches. Plant spacing for lettuce grown in deep water culture is the same as the spacing previously discussed for NFT systems. Deep water culture was experimentally shown to improve lettuce quality compared to drip irrigation or traditional soil methods [39]. This method reduced water use by 50% compared to traditional agriculture.

### 2.6. Aeroponics

**Figure 8:**
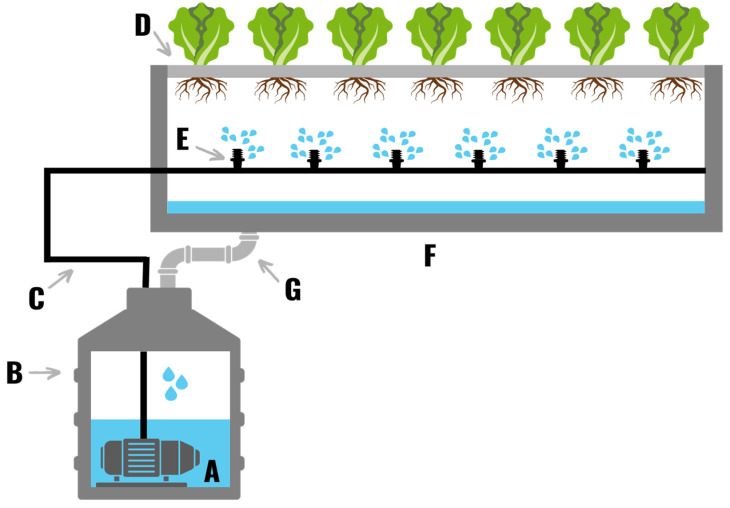
An aeroponic system consisting of a water pump (**A**), nutrient reservoir (**B**), watering line (**C**), a plant support plate (**D**), spray heads/nebulizers (**E**), a collection tray (**F**), and a return pipe (**G**). Plants are supported above the spray heads such that the roots of the plant are exposed to the air. Nutrient solution is misted onto the roots of the plants via the spray heads and runoff is collected and returned to the nutrient solution reservoir.

Aeroponic systems were invented in the 1980’s and consist of a pressure sprayer with a micro-inject nozzle/nebulizer that sprays nutrient solution in the form of a mist around the root zone of the plant from below (Figure 8). The plants are suspended above the nozzle in a platform similar to the fixed, suspended platforms used in some deep-water culture systems [16].

Aeroponic systems are the most technically difficult of all hydroponic systems to operate as there is a high potential for failure if an error occurs, as the roots of the plant are exposed in an aeroponic system. The misting schedule must be carefully calibrated for each type of plant to ensure that the roots do not dry out, and misting systems are easily negatively impacted by cool temperatures. Aeroponic systems are also expensive to install and require regular maintenance to ensure that spray heads remain unclogged [40]. Aeroponic systems are used by several commercial growers to produce small leafy plants and potato mini-tubers [19].

### 2.7. Aquaponics

**Figure 9:**
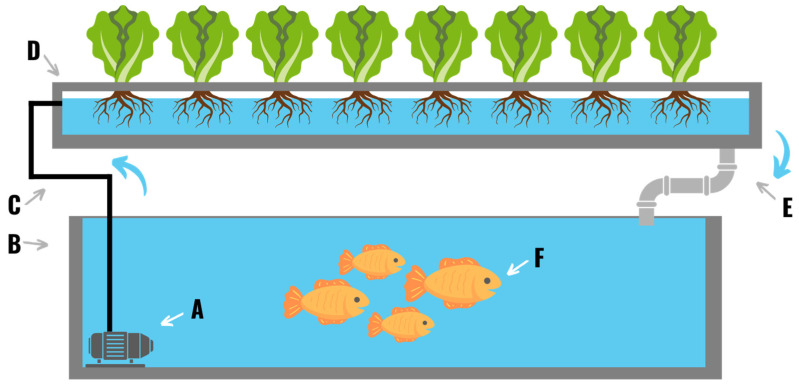
An Aquaponics system composed of a pump (**A**), fish tank (**B**), watering line (**C**), NFT troughs (**D**), water collection pipe (**E**), and fish (**F**). Fish and microbes in the fish tank generate effluent which is used as a nutrient source for the plants. The effluent water is pumped over the roots of the plants, which clean the water by removing metabolic byproducts that are toxic to the fish. The cleaned water is then returned to the fish tank.

Aquaponics is the practice of growing fish and plants in the same system. The earliest known applications of aquaponic methodology come from Southeast Asia around 5 CE where farmers cultivated rice and fish such as carp and eels in the same fields [41]. In the 1950’s researchers in Japan began developing more complicated recirculating cultivation systems to maximize food production from a fixed amount of space [41]. Modern aquaponic systems are a combination of one or more of the standard hydroponic system types and an aquaculture system (Figure 9). In this type of system fish excrement and microbial activity from the aquaculture system generate byproducts that are used by the plants as nutrients [42]. The plants then remove these byproducts from the water, thereby cleaning it, before it is returned to the fish tank [43]. Aquaponic systems are typically more complex than traditional hydroponic systems due to the need to balance system parameters to meet the needs of both the fish and the crops. Aquaculture systems can be either recirculating or non-recirculating depending on system design [41].

### 2.8. Substrates

In comparison to soil-based agriculture, hydroponic plants are grown in soilless substrates. Common soilless substrates include rockwool, coco coir (ground up coconut husk), peat moss, coconut fiber, perlite, vermiculite, and clay balls [19]. These substrates are inert, meaning that they provide little to no nutritional value to the plant and are primarily used as mechanical support for the plant or its root system [15]. With regard to hydroponic system design, plants are either grown with just enough substrate to provide a mass to support the plant in the system (for example deep water culture and NFT), or in larger quantities of substrate that contain all the roots of the plant (for example drip irrigation).

### 2.9. Nutrient Solution

Inorganic nutrient sources (also referred to as “chemical fertilizers”) are the most common source of nutrients used in hydroponic systems. The nutrient solutions used in a hydroponic system provide all 17 elements essential for plant growth. Growers can either add these elements individually or use a commercially available fertilizer mix [44]. Due to the increasing popularity of hydroponic crop production, there are many commercially available fertilizer mixes that have been designed specifically for use in hydroponics. Nutrient solutions suitable for hydroponic production dissolve completely and do not pose a clogging risk to drip lines.

To optimize growth, special care must be taken to maintain proper pH and electrical conductivity (EC). Optimal pH and EC values for the common hydroponically grown crops have been previously reported by Sharma and colleagues in 2018 [15] and for cannabis by Jin et al. in 2019 [45]. Optimal pH ranges are often between 5.0 to 7.0. EC values typically range from 1.0 to 3.0 dSm^-1^, although some crops such as beans and tomatoes have optimal values up to 4.0 dSm^-1^ [15]. High EC values indicate an overabundance of salts in the nutrient solution, which can prevent nutrient absorption by plants due to osmotic stress. Low EC values indicate insufficient nutrients in the system which can impair plant growth [43].

### 2.10. Lighting

Lighting is an essential aspect of optimizing plant growth, and as such it is an area of significant research with regard to controlled environment agriculture. Traditionally, growers implemented hydroponic systems inside of greenhouses to utilize natural sunlight for crop production. Glass was the original glazing material used in greenhouse construction and remains a popular choice to this day due to its light transmission properties. In the 1950’s polyethylene film was introduced as a glazing option, which enabled greenhouse production to expand outside of the developed world due to its lower cost. Rigid plastic materials were introduced in the 1990’s as a glazing option that provided increased light distribution (reduction of shade spots) in comparison to glass, with increased durability in comparison to plastic film [10].

Greenhouses in northern climates are often supplemented with artificial lighting during the winter months to maintain optimal plant growth. In modern greenhouses the two most common types of lights are high pressure sodium (HPS) and light emitting diode (LED) lamps. Despite their higher upfront cost, LEDs have gained popularity due to their long-life span, high energy efficiency, and low heat output [46].

LED’s have also enabled the rise of indoor agricultural systems that rely entirely on artificial lighting. Artificial lighting enables growers to further optimize growing parameters such as the wavelength and duration of light to maximize crop production. However, the cost of implementing and operating LED lighting systems can prove to be a major roadblock with regard to the profitability of indoor agriculture. As such, less expensive electricity sources and more efficient LEDs must be developed to improve the viability of this type of production.

## 3. Plant Growth-Promoting Bacteria

Although there are a wealth of hydroponic system options available for growers to select from, further advancements are needed to improve profitability via increases to crop yields. Hydroponic greenhouses need large capital investment to build, and require significant electricity and labour inputs to operate [47]. Fertilizers have significantly increased in cost in part due to increased global trade instability and supply disruptions. Indeed, prices increased 78.6% year-over-year in 2021 [48]. Moreover, there are several types of crops that are rarely profitable to grow hydroponically, including corn, potatoes, and large root vegetables (e.g., onion, carrots, and rutabaga). Solutions are needed that strengthen local food production and expand the range of viable hydroponic crops for growers to select from. Decades of research in soil suggest that microbial inoculants comprised of plant growth promoting bacteria constitute an exciting solution.

**Figure 10:**
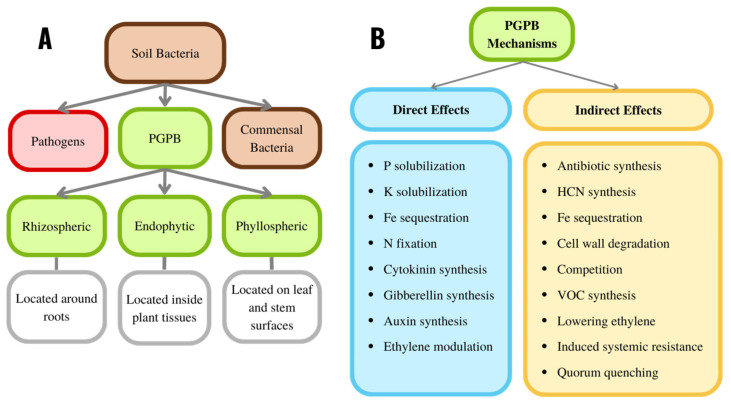
(**A**) Overview of some of the components of soil bacteria and their localization. (**B**) Overview of the major mechanisms used by PGPB. VOC refers to volatile organic compounds.

The interaction between soil bacteria and plants may be beneficial, harmful, or neutral for the plant. Beneficial plant growth-promoting bacteria (PGPB) facilitate plant growth by several different mechanisms. They are typically found in the soil along with bacteria that are deleterious to plant growth (phytopathogens) and bacteria that do not have any discernible effect on plant growth and development (commensal bacteria) (Figure 10A).

The soil contains a wide range of living entities including various microorganisms (bacteria, fungi, algae, and protozoa), nematodes, and earthworms with ~95% of these organisms being bacteria [49]. The number of bacteria that typically exist around the roots of plants is about 10- to 1000-fold greater than the number that are found in the bulk soil, with the highest concentration of bacteria being found immediately around the roots of plants in the rhizosphere [50]. The preponderance of microorganisms in the immediate vicinity of plant roots is a direct consequence of the fact that plants commonly exude a significant fraction of their photosynthetically fixed carbon through their roots [51,52]. Different plants produce varying amounts and compositions of root exudates; root exudates are also affected by plant age and nutrition as well as the presence of environmental stressors. Root exudates provide bacteria with carbon substrates that drive microbial metabolic processes. Each plant species (and subspecies) exudes specific small molecules so that plants “attract those microorganisms that are beneficial to plants and exclude those that are potentially pathogenic” [53]. Thus, the chemical composition of the root exudates of a particular plant species shapes the microbial community within the rhizosphere of that plant.

Many of the PGPB from the plant rhizosphere bind directly to the surface of the plant roots (i.e., they are found in the rhizoplane) (Figure 10A). Some PGPB can colonize the interior surfaces of the plant (i.e., they are found in the plant’s endosphere) without harming the plant. Other PGPB bind to the surface of plant leaves and stems (i.e., they are in the phyllosphere). Except for the determinants used by PGPB to localize rhizospherically, endophytically, or phyllospherically, all these bacteria utilize similar, if not identical, mechanisms for facilitating plant growth and development [54,55].

### 3.1. Mechanisms Used by PGPB

PGPB use a variety of mechanisms to positively impact the growth of plants including increasing plant biomass, nitrogen/phosphorus/potassium/iron content, root length, shoot length, seed germination, photosynthesis, resistance to the inhibitory effects of phytopathogens, ability to proliferate in the presence of various environmental stressors, and plant production of useful secondary metabolites. It is important to bear in mind that different PGPB utilize various mechanisms to promote plant growth. Moreover, any specific PGPB strain may use any of the multiple available mechanisms, either direct or indirect or a combination of the two, independent of the genus and species of the PGPB. Different plant species, plants of distinct developmental stages, plants grown in varying environments, and distinct cultivars of a plant species often respond uniquely to a specific PGPB strain and its mechanisms; this may reflect variations in plant physiology and/or biochemistry. These differences in response to a particular PGPB strain may also be a consequence of plant age and growth conditions including soil composition, plant growth temperature, and the presence or absence of stressful compounds and/or phytopathogens in the soil.

PGPB can positively affect plant growth and development employing either direct or indirect mechanisms [56] (Figure 10B). Direct mechanisms of plant growth promotion of plant growth include PGPB facilitating the acquisition of essential nutrient resources from the soil or air (e.g., phosphorus, potassium, iron, or fixed nitrogen). In other direct mechanisms of plant growth promotion, PGPB can regulate the concentration of phytohormones within a plant. For example, some PGPB directly affect plant growth by synthesizing the phytohormones auxin, cytokinin and gibberellin [57]. In addition, some PGPB can lower plant concentrations of the phytohormone ethylene by synthesizing the enzyme, 1-aminocyclopropane-1-carboxylate (ACC) deaminase, that cleaves the compound ACC which is the immediate precursor of ethylene in all higher plants.

Even though most soils contain relatively large amounts of phosphorus, most of the phosphorus is insoluble and therefore not available to support plant growth [58]. The phosphorus is present as either inorganic mineral forms such as apatite or as organic forms such as inositol phosphate. Moreover, a large percentage of the soluble inorganic phosphate that is applied to soils as a chemical fertilizer rapidly becomes insoluble following its application. Fortunately, many inorganic phosphates can be solubilized by PGPB-synthesized and secreted organic acids. The organic acids secreted by some PGPB also assist in releasing potassium from some insoluble minerals [59,60,61]. In addition, some PGPB secrete enzymes that can solubilize organic phosphates.

Despite the high concentrations of iron in most soils, ferric ion (Fe^+3^) is only sparingly soluble in water so that it is generally not present in most soils in sufficient amounts to support plant growth. Fortunately, many PGPB synthesize low molecular weight siderophore molecules that solubilize and chelate iron from the soil [62,63]. The iron-siderophore complex can be taken up by plants as well as PGPB thereby providing the plants (and PGPB) with a ready source of iron.

A large amount of energy is required to convert atmospheric nitrogen gas to ammonia, which is used by plants in the synthesis of proteins and nucleic acids. The chemical synthesis of ammonia requires a metal catalyst as well as very high temperature and pressure, depleting available non-renewable energy resources and causing environmental hazards. Fortunately, some soil bacteria can biologically fix gaseous nitrogen into ammonia using ATP as an energy source [64,65]. The best characterized nitrogen-fixing bacteria are Rhizobia, Gram-negative bacteria that form nodules on the roots of specific legumes. Within the root nodules, Rhizobia fix nitrogen and provide it to the plant while the plant provides low molecular weight carbon compounds (derived from photosynthesis) which fuel bacterial metabolism and nitrogen fixation. In addition to various strains of Rhizobia, several free-living bacteria can also fix nitrogen and provide it to plants, although somewhat less efficiently than Rhizobia.

A relatively small number of PGPB and other soil bacteria can synthesize cytokinins [66,67]. The rate limiting step in cytokinin biosynthesis is often catalyzed by the enzyme isopentenyl transferase (IPT) in both plants and bacteria, including phytopathogens and PGPB [68]. Cytokinins produced by microorganisms have had beneficial effects on plants via multiple pathways, including pathogen inhibition and drought tolerance. Researchers have shown that cytokinin-producing *Agrobacterium* strains can prime tobacco immune defenses against the pathogen *Pseudomonas syringae* [69]. Of interest, the cytokinin dose was critical in determining the activation level of mitogen-activated protein kinase (MAPK) pathway genes. When levels exceeded an optimal threshold a decrease in MAPK activation was observed. A similar result was recorded when *Pseudomonas fluorescens* reduced *P. syringae* infections in *Arabidopsis* due to cytokinin production [70]. Recent research in *Arabidopsis* demonstrated that the cytokinin-producing fungi *Trichoderma* can also reduce the severity of *Fusarium* infections [71]. Under drought conditions, a *Bacillus subtilis* species significantly increased shoot cytokinin concentrations by ~30% and increased conifer leaf health in *Platycladus orientalis* [72], whereas *P. fluorescens* cytokine production improved drought tolerance in tomatoes [73].

Like cytokinins, it appears that only a small number of PGPB synthesize the plant hormone gibberellin [74]. Nevertheless, a very large number of different gibberellin molecules have been identified, although, except for the gibberellin molecule GA3, the biological activity and role of most other gibberellins is unclear. This notwithstanding, gibberellins have been shown to be involved in increasing plant stem growth, germination dormancy, flowering, and leaf and fruit senescence [75].

The positive effects of PGPB on plant growth are often explained by the bacterial synthesis of the phytohormone auxin [57,76,77,78,79] with indole-3-acetic acid (IAA) being the most studied and likely the most common auxin molecule. Some precursors and derivatives of IAA also have auxin activity. In addition, several synthetic auxins have been used in specialized applications and are commercially available [80].

Auxins play a key role in the response of root and shoot growth to light and gravity, differentiation of vascular tissue, apical dominance, initiation of lateral and adventitious roots, stimulation of cell division and elongation of stems and roots. In addition, by loosening plant root cell walls, IAA can increase the amount and alter the type of molecules present in root exudes thereby selecting for different plant rhizospheric bacteria. Overall, the PGPB and plant auxin concentration are central to a plant’s response to a wide range of environmental factors. Different plants (including different cultivars of the same plant) as well as different plant tissues are sensitive to dramatically different levels of auxin. Thus, for example, the optimal concentration of auxin that is needed for promoting plant tissue growth is approximately five orders of magnitude higher for leaves and shoots than it is for roots. The endogenous level of IAA that exists within a plant is critical in determining whether additional (bacterial) IAA will stimulate or inhibit plant growth [81]. Thus, exogenous bacterial IAA may alter the level of IAA within a plant tissue to either an optimal or supraoptimal amount. Since most (~85%) rhizospheric bacteria synthesize IAA, it is likely that in the absence of IAA producing bacteria, the IAA level in many plants is generally suboptimal for maximal plant growth and development.

The phytohormone ethylene which is synthesized in all higher plants catalyzes many different biological activities. It is active over a very wide range of concentrations, from 0.05 μL/L to 200 μL/L [82]. Ethylene impacts seed germination, tissue differentiation, the formation of root and shoot primordia, root branching and elongation, lateral bud development, flowering initiation, anthocyanin synthesis, flower opening and senescence, fruit ripening and degreening, production of volatile organic compounds responsible for aroma formation in fruits, storage product hydrolysis, leaf senescence, leaf and fruit abscission, Rhizobia nodule formation, mycorrhizae-plant interaction, and the response of plants to biotic and abiotic stresses [82].

The enzyme ACC deaminase (found in many soil bacteria and some soil fungi) cleaves the molecule 1-aminocyclopropane-1-carboxylate (ACC), the immediate precursor of ethylene in higher plants, to ammonia and α-ketobutyrate. Modulation of a plant’s ACC content, and hence its ethylene content, is one of the central mechanisms used by PGPB to facilitate plant growth. ACC deaminase, which is synthesized by many PGPB, lowers the amount of ethylene inhibition of plant growth that occurs because of various environmental stresses [83,84].

Following the binding of PGPB to a plant surface (usually roots) the bacteria generally synthesize IAA which is taken up by the plant, and both stimulates plant growth and induces the transcription of plant encoded ACC synthase, which converts plant S-adenosyl methionine to ACC [85]. The IAA also increases plant root exudation thereby providing a portion of the plant’s ACC to the plant surface bound PGPB, which take up the ACC before cleaving and metabolizing it. This results in the concentration of ACC inside plant tissues being lowered as the plant bound bacterium acts as a sink for ACC. Therefore, the level of plant synthesized ethylene is decreased and there is subsequently less ethylene inhibition of plant growth. This protects plants from increased ethylene concentrations that typically follow environmental stresses. Decreasing a plant’s ACC concentration lowers the ethylene inhibition of the plant’s IAA signaling pathway thereby allowing bacterial IAA to better promote plant growth. This series of events is beneficial to the growth of a wide range of plants [86].

The indirect promotion of plant growth occurs when a PGPB prevents, or decreases, the damage to plants that might otherwise ensue because of infection of the plant by phytopathogens [23,49]. The phytopathogens typically include various soil fungi and bacteria. In addition, biocontrol PGPB, using a range of different indirect mechanisms, may lessen the damage to plants from either insects or nematodes [87]. The indirect mechanisms employed by PGPB to promote plant growth include the synthesis of antibiotics that inhibit the growth of (mostly fungal) phytopathogens; the synthesis of hydrogen cyanide that acts as an adjunct to antibiotic inhibition of fungal growth; the synthesis of bacterial siderophores which prevent phytopathogens from acquiring sufficient iron for their proliferation; the synthesis of enzymes such as glucanases and chitinases that can break down fungal (and some insect larval) cell walls; outcompeting phytopathogens with beneficial organisms; synthesis of small volatile organic compounds (VOC) that are toxic to many phytopathogens; synthesis of ACC deaminase which lowers plant stress ethylene levels caused by phytopathogens that might otherwise decrease plant growth; turning on plant induced systemic resistance (ISR) which is a plant defense against phytopathogens which needs to be activated; and synthesis of quorum quenching enzymes that prevent many phytopathogens from reaching a high density (Figure 10B).

### 3.2. Bacterial Consortia

For the most part, PGPB have been studied as single isolates functioning under laboratory conditions. However, in nature, plant roots are surrounded by a multiplicity of bacteria, and in these root microbiomes bacteria act in a concerted manner to influence plant growth and development [53,55,88,89]. In a limited number of instances to date, scientists have been able to combine several individual PGPB which act as a small PGPB consortium that can promote plant growth and development. While there are many literature reports elaborating the many different bacteria found in the microbiomes of various plants under different conditions [53] at present scientists have little understanding of how to artificially construct functional and effective bacterial consortia that are stable and are optimized to promote plant growth.

## 4. Plant Growth Promoting Bacteria Research in Hydroponics

### 4.1. The Hydroponic Microbiome

The Dutch botanist and microbiologist Lourens Bass-Becking once said, “Everything is everywhere, the environment selects”. Given that a hydroponic system is distinct from a soil environment surrounding the rhizosphere, it stands to reason that the same plant variety may have different rhizospheric microbial communities depending on whether it is growing in soil or a soilless environment. Hydroponic systems have different levels of moisture, oxygen [90], and nutrients [91] than soil. Oxygen exchange is highly dependent on the matrix being used: soil oxygen and moisture levels affect biogeochemical cycles and the nitrogen cycle [92]. These systems can result in changes to the crop quality. For example, hydroponic lettuce had a stronger root system and more moisture content than a soil-grown crop, although antioxidant content was reduced [93]. Barley was more susceptible to salt stress in hydroponic systems compared to soil-based plants [94]. Several research groups have conducted thorough studies to obtain empirical data on how differences between soil and soilless environments affects not just crops but the hydroponic microbiome.

An important question is whether hydroponic systems have elevated levels of human pathogens [95,96]. Every year large recalls occur due to contaminated produce, thus a growing environment that is low in pathogens is critical if the amount of produce grown in hydroponics continues to increase as projected. Human pathogens are uncommon native organisms in hydroponic systems, further supporting the safety of this agricultural method for consumers. For example, *Clostridium botulinum*, *Escherichia coli*, *Salmonella*, and *Staphylococcus aureus* were not found within lettuce hydroponic systems [95]. Water and leaves from hydroponically grown lettuce in Puerto Rico were analyzed to characterize potential human pathogens [96]. *Entercoccus faecalis* was the most predominant pathogen, found in 11% of leaf samples, but not in the water. A range of 4–70 CFU/mL total bacteria were quantified in leaves, which was lower than >300 total CFU/mL found in the water system. Detectable levels of *E. coli* O157:H7 and *Salmonella* were not observed within the samples. However, ~78% of their samples contained bacterial isolates, including *Aeromonas*, *Bacillus*, *Corynebacterium*, *Mycobacterium*, *Pediococcus*, *Pseudomonas*, *and Serratia* [96]; many of these bacteria are plant growth promoters in hydroponic systems (Supplementary Table S1).

High throughput sequencing has enabled scientists to ascertain which organisms are prevalent in hydroponic systems. A study of the influence of urine-derived fertilizers conveyed the range of OTUs that are native to the hydroponic lettuce rhizosphere [97]. *Pseudomonas* was the only genus that was a true indicator organism, present in over 90% of samples. *Burkholderia* and *Sphinogomonas* were also highly prevalent. Out of 185 identified OTUs, other highly indicative families were *Rhizobiaceae*, *Chitinophagaceae*, and *Flavobacteriaceae*. Hydroponic derived organisms found surviving in the plant rhizosphere are more likely to persist in a hydroponic system than soil-derived organisms. This study did not conclude that *Bacillus* was an indicator organism in hydroponics, which is a notable difference from soil studies of the same crops. Anzalone et al. [24] conducted a tomato rhizosphere metagenomics study comparing the communities between soil and soilless coconut fiber environments. They concluded that the tomato microbiome was controlled by the environment in which the plant grew. Significant differences in microbial communities were observed in soil vs. hydroponic systems. The hydroponic tomato rhizosphere had significantly reduced bacterial and fungal diversity, despite coming from identical nursery stock [24]. PCoA plots visualizing community similarities clearly showed that samples grouped by substrate type. These findings suggest that organisms isolated from soil may not always be able to survive on the same plant in hydroponic greenhouses.

A taxonomic survey was conducted in a lettuce hydroponic facility to determine the microbial communities present in the water, nutrient solution sump, biofilter effluent sump, and tilapia aquaculture tanks [98]. The plants had a strong influence on the microbial community present, which remained relatively constant despite various treatments. They concluded that the impact of microbial inoculants on the community structure was lower than expected and suggested that growers and scientists need to carefully balance sterilization vs. the need to maintain a healthy microbiome in the systems. However, it must be noted that the organism used to formulate these conclusions was *Bacillus amyloliquefaciens*, supporting evidence that *Bacillus* species do not always perform as well as other organisms, such as *Pseudomonas*, that thrive in hydroponic systems.

A study by Sheridan and colleagues analyzed microbial community changes of potato, soybean, durum wheat, and bread wheat crops after receiving a commercial microbial inoculant containing 48 strains of different organisms [99]. The authors determined that the most abundant organisms in the mix did not correlate with the most effective colonizer in a hydroponic system. They observed that microbial communities were specific to the crop type, indicating the same mixture does not interact to the same extent with all crops that were tested. Interestingly, an unexpected ten-hour 50 °C heat event in the durum wheat hydroponic system caused a shift in the crop’s microbiome, resulting in the thermophile *Chlorobi* OPB56 significantly increasing in abundance. As the planet warms and heat events become more frequent, the knowledge that heat shifts can change the hydroponic rhizosphere is important. A separate study that focused on which traits created the best hydroponic colonizers concluded that better PGPB colonizers of Duckweed roots contained relatively more genes for bacterial chemotaxis, flagellar assembly, and two-component systems [100]. This suggests that the ability for a bacterium to travel and move towards plant exudates increases its ability to colonize hydroponic roots.

### 4.2. PGPB That Increase Nutrient Uptake

Plants need a range of macro and micronutrients to thrive. The six macronutrients are carbon, hydrogen, nitrogen, oxygen, phosphorus, and potassium while the eight plant micronutrients are boron, chlorine, copper, iron, manganese, molybdenum, nickel, and zinc. PGPB that increase nutrient uptake for crops improve the availability of one or more of these nutrients to facilitate plant growth. Increasing nitrogen, phosphorus, and iron uptake are the most commonly tested strategies in the hydroponic PGPB literature.

Nitrogen is an essential plant nutrient that is critical for amino acid synthesis, chlorophyll, and nucleic acid development. Plants cannot obtain nitrogen directly from the atmosphere, thus relying on alternative forms such as ammonia and nitrate. Nitrogen is one of the main components of chemical fertilizers. Globally, approximately 115 million tons of nitrogen is applied annually to fields [101], although only a third of this is taken up by the plants. Bacteria participate in the nitrogen cycle converting gaseous nitrogen into more available forms. Nitrogen fixation converts atmospheric N_2_ into ammonia; ammonia oxidation then converts ammonia into nitrite, which can then be converted into nitrate via nitrite oxidation. Nitrogen fixing bacteria include *Rhizobia*, *Azospirillum*, *Azotobacter*, *Bacillus*, and *Beijerinckia*. When added to a nitrogen-free hydroponic system, *Azospirillum* and *Bacillus* increased nitrogen yield in bananas by up to 144%, shoot growth by ~200%, and biomass by ~140% [102]. In one study, *Azotobacter* was immobilized onto beads. The author determined that adding 5g of beads per plant optimized growth of Choy sum (a Chinese flowering cabbage) [103]. The presence of *Acinetobacter* increased the amount of nitrogen that Duckweed could obtain from pondwater [104]. A commercial mixture of *Bacillus* spp. influenced the levels of ammonia, nitrite, and nitrate in an NFT system containing Red Cherokee lettuce [105]. A second lettuce study using Tiberius romaine lettuce developed a consortium containing multiple nitrogen fixers, including *Azotobacter chroococcum*, *Azospirillum brasilense*, *Pseudomonas fluorescens*, and *Bacillus subtilis* [106]. The authors observed that the amount of nitrogen uptake almost doubled, especially when (non-nitrogen-fixing) arbuscular mycorrhizal fungi were also added in with the bacteria. Lastly, a mixed culture of unspecified nitrifiers and ammonifiers was able to utilize organic nitrogen in a tomato crop [107], creating the potential for hydroponic growers to switch to organic fertilizers instead of the typical inorganic fertilizers.

Phosphorus is another macronutrient that is essential for plant health. It is a building block of nucleic acids, and contributes to photosynthesis, root growth, and maturation. Phosphorus solubilizing bacteria convert phosphorus from the nutrient solution into a more bioavailable form, as plants can only absorb phosphorus in monobasic and dibasic forms. Similar to its involvement with the nitrogen cycle, *Acinetobacter* increased the amount of phosphorus that duckweed could obtain from pondwater [100]. A mixture of *Bacillus* spp. increased phosphorus solubilization for lettuce and increased yields [105] PGPB identified in a sorghum trial included a range of the phosphorus solubilizing bacteria *Pseudomonas* spp., *Burkholderia* spp., *Phylobacterium* spp., and *Chitinophaga japonensis* [108]. Phosphate solubilization is a common trait of *Pseudomonas* spp. in switchgrass [109,110], and tomatoes [111,112]. Several beneficial organisms were found to have both nitrogen fixation and phosphorus solubility, including *Pantoea agglomerans* in rice experiments [113]. An experiment with soybeans evaluated the increase in photosynthesis capabilities after applying a commercial microbial consortium [114]. The complex mixture of bacteria, yeasts, and fungi was hypothesized to be providing multiple benefits to the soybeans, including improved nitrogen and phosphorus uptake.

The micronutrient iron is a key component of chlorophyll, and as such iron deficient plants undergo chlorosis, which is characterized by yellow leaves from a lack of chlorophyll. Iron also improves plant enzymatic functions and respiration. Iron uptake is increased by bacteria that secrete siderophores, which are high-affinity iron chelators that effectively bind iron and increase iron sequestration for both the plants and bacteria. A study in canola focused on four PGPB that all possessed siderophores amongst other beneficial traits [115]. *Arthrobacter*, *Bacillus altitudinis*, *Bacillus megaterium*, and *Sphingomonas* increased biomass production; however, the authors concluded that *Sphingomonas* was the best candidate for future studies. A cucumber trial that tested the beneficial fungus *Trichoderma harzianum* resulted in inoculated plants possessing an increase in multiple nutrients including phosphorus, iron, copper, manganese, and zinc [116]. Although the authors did not test for siderophores, they observed a 90% increase in phosphorus and 30% increase in iron within the plants. More recent advances have shown that *T. harzianium* produces a novel siderophore called harzianic acid [117]. Interestingly, a strawberry study demonstrated that not all siderophore structures behave equally [118]. They concluded that bacteria with hydroxamate siderophores produced by the PGPB *Gluconacetobacter diazotrophicus* were more beneficial for iron uptake to the crop than catechol siderophores produced by *Azospirillum brasilense*. Catechols are less stable than hydroxymates and are susceptible to oxidation.

### 4.3. PGPB That Regulate Hormones

Control of the phytohormone ethylene via ACC deaminase is an effective strategy to increase crop yields in hydroponic systems. PGPB with this gene have been isolated from the international space station [119]. When twenty bacterial species were analyzed for a range of PGPB traits it was determined that *Pseudomonas agglomerans* and *Bacillus pyrocinnia* both possessed multiple PGPB beneficial traits, including ACC deaminase, phosphate solubilization, and siderophore production. A study that observed over 20% increases in canola yields also used bacteria that possessed functional ACC deaminase, IAA, phosphate solubilization, and siderophores [115]. Likewise, the best strain in terms of promoting plant growth out of 305 isolates in rice experiments possessed ACC deaminase, IAA, and siderophores [120]. Additionally, bacteria with a range of beneficial traits including ACC deaminase, IAA, phosphorus solubilization, and N cycling increased wheat yields [121]. These studies highlight that single strains that possess at least three functional plant growth promoting traits including ACC deaminase are highly successful in increasing yields in a wide range of crops.

Bacterial IAA is an auxin involved in L-tryptophan metabolism that is responsible for increases in plant growth. Within cucumbers, the two most successful PGPB tested, *Serratia marcescens* and *Pseudomonas putida*, were also the two strains that produced IAA [122]. Both organisms performed better than *Bacillus amyloliquefaciens* and an unspecified *Bacillus* spp. strain 70. Consortium trials of five IAA producers increased wheat yields from 36–80% [123]. A consortium of auxin producers including *Bacillus cereus*, *Bacillus thuringiensis*, and *Buttiaxella agrestis* remarkably reduced the time required for banana seedling acclimatization from 90 to 25 days [124]. Many PGPB have multiple beneficial traits, as evidenced by this consortium’s ability to also produce the hormone cytokinin, hydrocyanic acid, siderophores, and solubilize phosphorus. A lettuce trial using *Gluconacetobacter diazotrophicus* observed up to 16% increases in yield using an organism that produces both the hormones IAA and gibberellin [125]. Similarly, bacteria possessing IAA, nitrogen fixation, and phosphorus solubilization increased rice yields up to 20% [113,126]. *Pseudomonas fluorescens* increased tomato crop yields by up to 18% [127]. Although the authors were unsure of the exact mechanism, they suspected growth regulating substances were involved. Many strains of *Pseudomonas fluorescens* have been documented as having both IAA and ACC deaminase in more recent research [128]. Indeed, IAA and ACC deaminase producing *Pseudomonas* outperformed other isolates in sorghum [108], switchgrass [109,110], and tomato trials [111]. Single organisms with the ability to produce both hormones and increase nutrient uptake frequently perform the best in both soil and hydroponic systems [86].

### 4.4. Biocontrol Agents

Crop diseases significantly harm global food production and can have a devastating effect in greenhouses that become contaminated. Implementing biocontrol efforts to reduce the severity of infections are an effective strategy to increase yields in hydroponic farms. A wide range of PGPB have been tested against hydroponic phytopathogens.

*Pythium* is a parasitic oomycete that causes root rot and damping off in many crops including ornamental flowers, arugula, cucumber, lettuce, spinach, sweet pepper, and tomato [129]. A study of hydroponically grown Chrysanthemums concluded that *Pseudomonas chlororaphis* and *Bacillus cereus* were the best PGPB for *Pythium* biocontrol after they reduced pathogen root colonization by 72–91% [130]. *Pseudomonas chlororaphis* also effectively prevented *Pythium* infection in Romaine lettuce [16] and Cubico sweet peppers [131]. In Cortina lettuce, the commercial product “Boost” containing *Bacillus subtilis* was more effective than other products with *Enterobacter*, *Trichoderma*, or *Gliocladium* [132]. The mechanism of action was unknown, but the authors suspected that the organisms were inducing plant resistance or preventing *Pythium* colonization. Their conclusions were further supported by a separate study that observed that *Bacillus* also reduced *Pythium* root colonization in a trial with Red Coral and Green Oak lettuce [133]. A hydroponic tomato study observed >50% decrease in disease incidence when either *Pseudomonas fluorescens*, *Gliocladium*, *Trichoderma*, or *Streptomyces* were applied [134]. Applying *Lysobacter enzymogenes* in combination with chitosan reduced disease in cucumber plants by 50–100% in four independent trials [135]. Another cucumber trial tested four commercial inoculants to determine their efficacy against *Pythium* [136]. Mixtures containing either *Gliocladium catenulatum* or *Streptomyces griseoviridis* were more effective than *Trichoderma* treatments. Together, these studies suggest that *Pseudomonas chlororaphis*, *Bacillus* spp., and *Gliocladium* work effectively in a range of crops to prevent root rot and damping off. Indeed, the body of literature suggests that *Bacillus* is more effective at preventing *Pythium* infections than it was at colonizing hydroponic roots and increasing growth in healthy non-infected plants.

Several other plant diseases are relevant to hydroponic systems and may be alleviated by inoculating crops with PGPB and fungi as a means of biocontrol. *Fusarium* is a filamentous fungus that causes wilt disease in a wide range of crops. Several researchers have studied PGPB in an attempt to find suitable biocontrol candidates. Like biocontrol strategies for *Pythium*, the bacteria *Gliocladium catenulatum* and *Pseudomonas chlororaphis* significantly reduced *Fusarium* seedling mortality [137]. Commercial mixtures containing *Trichoderma* or *Streptomyces* reduced disease incidence by >50% [134]. The pathogen *Ralstonia* is the causative agent of bacterial wilt disease. In one study, a combination of *Trichoderma viride*, *Bacillus thuringiensis*, and *Pseudomonas fluorescens* inhibited ~70% of bacterial wilt in Linda lettuce [138]. Another bacterial phytopathogen is *Pectobacterium* (formerly *Erwinia*), which causes vegetable soft rot; the bacterium *Rhodococcus* reduced maceration in potatoes by degrading the quorum sensing capabilities of the pathogen [139]. Lastly, *Colletotrichum graminicola* is a fungus that causes stalk rot in grains and maize. *Trichoderma virens* protected maize against *C. graminicola* infection and reduced disease severity. The *T. virens* inoculant produced Sm1, a compound that induced plant defense mechanisms [140].

### 4.5. Bioremediation and Osmotic Stress

Bacteria isolated from hydroponic crops have promising capabilities for bioremediation. Carbendazim is a fungicide that negatively impacts aquatic organisms. A biofilm consortium comprised of *Flavobacterium*, *Flectobacillus*, *Klebsiella*, and *Stenotrophomonas* was able to degrade ~35 mg/L carbendazim to ~8 mg/L in 20 h [141]. Two studies on switchgrass demonstrated that *Pseudomonas* species could reduce cadmium stress and increase plant growth in the presence of 20 µmol/L cadmium. The organisms also had a range of beneficial plant growth promoting traits including ACC deaminase, IAA production, and phosphorus solubilization [109,110]. Inoculated plants had elevated expression of the heat shock proteins HSP70 and HMA3, which improves cadmium tolerance in plants. A strain of *Pseudomonas fluorescens* also promotes cadmium uptake in the perennial plant *Sedum alfredii* [128], while *Pantoea agglomerans* reduced cadmium concentrations and increased yields by ~20% in rice [113]. Another contaminant that is toxic to plants and harms humans upon ingestion is arsenic [142]. Research in rice concluded that a combination of *Pseudomonas stutzeri* and *Cupriavidus taiwanensis* reduced arsenic toxicity in rice [126] by converting arsenic to a harmless arsenic sulfide form. The bacteria also had a range of beneficial plant growth promotion mechanisms including ACC deaminase, IAA, phosphorus solubilization, and nitrogen fixation.

Salinity stress can occur in hydroponic systems and reduce crop yields. A wide range of negative effects including reduced photosynthesis, reduced root elongation, stem diameter and plant height are a consequence of salinity stress [143]. In one study, each EC unit increase in salinity resulted in a 7.2% decrease in tomato yield [144]. Thus, adding PGPB into hydroponics to reduce these negative effects would significantly benefit growers. Some *Pseudomonas* strains increased osmotically stressed plant crop yields in canola by 10% [145]. The commercial inoculant TNC Bactorr consisting of *Bacillus* spp. and *Paenibacillus polymyxa* alleviated 20mM salt stress in Crispa variety lettuce [146]. Four treatments with this inoculant prevented the 15% yield decrease caused by the osmotic stress. The authors noted that the autumn harvest tolerated salinity stress better than in the spring. Multiple PGPB have reduced salt stress in rice [120,147]. *Bacillus amyloliquefaciens* increased yields by 15% in the presence of 200 mM salt, while upregulating 14 plant genes [147]. The authors noted that salt-stressed rice underwent a shift in the microbiome that enriched for organisms that produced osmoprotectants including trehalose. Trehalose biosynthesis was also a key mechanism in a stressed hydroponic tomato trial [148]. A study of 305 strains concluded that strain TY0307 (taxonomy undeclared) improved yields by 30% by reducing ROS stress, increasing proline concentrations, and producing ACC deaminase [120]. Lastly, a comprehensive study in wheat tested 18 bacterial strains in four salt concentrations to determine the best PGPB for salt stress [121]. The ~58% crop reduction under salt stress was decreased to ~15% when PGPB were added due to a range of beneficial traits including ACC deaminase, phosphorus solubilization, IAA, and N fixation. The most effective organisms tested were *Thalassobacillus*, *Bacillus*, *Halomonas*, *Oceanobacillus*, *Zhihengliuella*, and *Staphylococcus succinus*.

## 5. Future Directions

The employment of plant growth promoting bacteria in hydroponic systems is at an exciting stage. Researchers now have access to a plethora of different hydroponic systems, lighting options, and plant varieties to design high impact experiments. The reduced cost and speed of high throughput sequencing means scientists can now elucidate which organisms are in their systems. After reviewing the current hydroponics body of literature, we have several recommendations that would elevate the quality of research in the field.

It was observed that many of the articles did not conduct any mechanistic studies to explain why their organisms helped increase crop yields. The authors of these studies would often infer that a plant growth promoting trait such as ACC deaminase, IAA, phosphorus solubility, nitrogen cycle involvement, or siderophores were involved based on the bacterial taxonomy. The majority of these tests are inexpensive traditional microbiology culturing protocols and would improve the quality of future hydroponic research. Bacteria within the same family or genus can have varying expressions of these genes. Some organisms can also have a copy of a plant growth promotion gene; however, it may be inactive [149]. Therefore, sequencing the isolate alone is not sufficient to prove that a trait is biologically active. Additionally, even though a gene is present and active under laboratory circumstances does not mean that this trait is responsible for growth promotion within a particular environment. We encourage all research labs conducting studies into plant growth promoting microbes to conduct knockout mutation studies on the best growth promoting strains to determine specifically which traits are responsible for the positive phenotypes observed. The results from knockout mutation studies would provide compelling evidence that researchers could use to increase the robustness of their final conclusions.

A conundrum that researchers must account for is the level of biological variation that occurs from churlish plants that receive identical nutrition, lighting, and hydration. Despite best efforts to provide identical inputs, plants in the same treatment group will have a range of biomass and fruit production. The best way to mitigate this problem is for researchers to maximize the number of replicates. While many studies included excellent randomized block designs (Supplementary Table S1), maximizing the number of replicates as well as repeating the experiment in biological triplicate would ensure the best PGPB is selected for commercialization. Studies that include high quality images of their hydroponic setups enable readers to both observe the experimental design as well as evaluate biological variation, visible meaningful yield increases, and visually ascertain the health of the crops. Lastly, studies that analyze fruit bearing crops should also measure metrics for the portion of the plant that is commercialized, and not just shoot weight.

These studies demonstrate that when it pertains to microbial diversity, less was sometimes more. Hydroponic systems have lower diversity than soil-based environments. Studies using a single organism with multiple plant growth promoting traits or small consortia often had significant increases to growth yields (Supplementary Table S1). Although a subset of experiments utilized complex consortia, the presented data did not always clearly support the conclusion that every organism in the mixture was colonizing the roots and persisting. Many consortia formulations should be tested during development to ensure the organisms are persisting in the hydroponic system and to avoid antagonistic effects between organisms that are more effective as individual strains. When designing consortia, larger consortia formulations should be tested with enough smaller iterations to confirm that every strain is an integral component of the increase in crop yield. Researchers are encouraged to ascertain every organism in the mixture can survive in a hydroponic system, as the variations in oxygen, moisture, and nutrient content does not guarantee all microorganisms isolated from soil can survive in hydroponics. Additionally, excellent research has been conducted on a range of crops, however few of these beneficial PGPB have undergone the next step into commercialization and use in non-research greenhouses. Researchers are encouraged to collaborate so that the most effective organisms can transition from academia to growers and eventually consumers.

An intriguing area of future hydroponic PGPB research occurs when one recognizes that the sky is not the limit. Hydroponic systems are currently used in food production on the international space station [119] and are being planned for the lunar base Artemis program [150]. However, research advances are required to reduce the plant stress levels, chlorosis, and leaf curling that have been observed on the International Space Station (ISS) with red romaine lettuce and zinnias [151]. The use of PGPB constitutes one solution to this problem. Despite their small size, these microorganisms have enormous potential to improve crop yields both on this planet and beyond.

When it comes to feeding the growing human population hydroponic crop production shows significant promise. The reduced water requirements, lower fertilizer use, higher yields, and minimal geographic restrictions on construction offer several benefits. First, hydroponic farms can be constructed inside of cities and used to reduce food deserts in densely populated urban areas [152]. Second, the higher yields and lower land use requirements open up opportunities for the conversion of agricultural land back into native environments, thereby increasing global biodiversity and carbon sequestration. Thirdly, the lower fertilizer and water requirements and increased consistency of production offer a way for countries to increase food security. As improvements are made to both reduce costs and improve yields, hydroponic production has the potential to replace conventional production for many crop types. Additionally, as weather patterns continue to become more erratic there will be an increasing shift to controlled environment agriculture to meet food security requirements.

The use of PGPB in hydroponic production has the potential to further increase yields, environmental sustainability, and food security. In this review we have shown that a range of strategies have successfully been used to increase hydroponic crop yields, including reducing stress via ACC deaminase, increasing nutrient availability via phosphorus solubility, siderophores, and nitrogen fixation, as well as biocontrol of crop diseases such as *Pythium*. Going forward, a focus on designing robust, repeatable experiments will ensure the best PGPB are identified to help feed the growing human population.

## Data Availability

Not applicable.

## Supplementary Materials

The following supporting information can be downloaded at: https://www.mdpi.com/article/10.3390/plants11202783/s1, Table S1: Plant growth promoting bacteria studied in hydroponic systems.
