# Supplementary material for: The Use of PGPB to Promote Plant Hydroponic Growth
Source: Plants (Basel). 2022 Oct 20;11(20):2783. doi: 10.3390/plants11202783 (PMC9611108; doi:10.3390/plants11202783)
Supplement: Supplementary file 1 [file plants-11-02783-s001.zip › plants-1947526-supplementary.pdf]

Supplemental Table S1: Plant growth promoting bacteria studied in hydroponic systems

Crop	PGPB	Increase in yield obtained (%)	Number of plants tested	Day harvested	Hydroponic system used	Mechanism of action and main conclusion	Reference
Banana<br><br>Barangan dessert	• *Azospirillum*<br>• *Bacillus sphaericus*	137-141%<br><br>(nitrogen free system)	Randomized design<br><br>6 replicates<br><br>3 treatments	45 days post transplant<br><br>10-11cm height	Nitrogen free system<br><br>4L pot	• Nitrogen fixation from bacteria in a nitrogen free system<br>• Increased nitrogen uptake led to increased photosynthesis	Mia, 2010 [102]
Banana<br><br>BRS Princesa<br><br>Prata Anã	• *Bacillus cereus*<br>• *Bacillus thuringiensis*<br>• *Buttiauxella agrestis*	10.8 (BRS) - 31.7% (PA)	Randomized design<br><br>5 replicates, 3 plants<br><br>4 inoculants + control	90 days post transplant	5 floating systems<br><br>500 L reservoir<br><br>Daily nutrient replenishing	• Seedling acclimatization period was reduced from 90 to either 25 (PA) or 15 (BRS) days<br>• Auxin production<br>• Increased photosynthetic rate	Araújo, 2022 [124]
Canola<br><br>Sarigol	• *Pseudomonas fluorescens*	~10%	Split plots design<br><br>3 replicates	"Latest stage of morphological growth"	Unspecified system with 10-20L tanks	• Inoculated plants performed better under both regular and osmotically stressed (PEG 6000 - 50-70% FC) conditions<br>• Higher metabolic/energy related protein expression in inoculated plants	Gharelo, 2016 [145]

Canola<br><br>Xikouhuazi	• *Arthrobacter* sp.<br>• *Bacillus altitudinis*<br>• *Bacillus megaterium*<br>• *Sphingomonas*	10-20%	4 inoculants + control<br><br>6 replicates	3 months	Unknown	• ACC deaminase, IAA, phosphorus solubilization, siderophores	Pan, 2017 [115]
Choy sum	• *Azotobacter*	Percent yield increase not stated	Randomized factorial design<br><br>5 levels of PBPR concentration, 6 levels of IAA solution<br><br>5 pots per treatment	45 days	Sterilized pots containing vermiculite	• 5 g of immobilized *Azotobacter* sp. bead per plant and 1 ml/plant/week of 40-60 µM IAA solution was optimal for growth increases	Ma-on, 2009 [103]
Chrysanthemum<br><br>Fina	• *Pseudomonas chlororaphis*<br>• *Pseudomonas corrugata*<br>• *Pseudomonas fluorescens*<br>• *Bacillus cereus*<br>• *Comamonas acidovorans*<br>• *Burkholderia gladioli*	*Pythium* root colonization reduced 72-91%	Randomized complete block<br><br>Untreated+ pathogen controls<br><br>13 inoculants<br><br>4 plants/ exp Repeated twice	18 days	500mL or 1.9L individual units<br><br>Aerated via continuous bubbling	• *Pseudomonas chlororaphis* and *Bacillus cereus* were the best at *Pythium* biocontrol	Liu, 2007 [130]

Cucumber	• *Pseudomonas putida*<br>• *Serratia marcescens*<br>• *Bacillus spp.*<br>• *Pseudomonas fluorescens*<br><br>Commercial product:<br>• *Bacillus amyloliquefaciens*	78.5-121.1%<br><br>Commercial product was 66.1% increase	Randomized block<br><br>5 PGPR +control<br><br>9 plants/pot<br><br>4 replicates	14 weeks	Drip irrigation<br><br>2L/h flow rate<br><br>8L Perlite/plant<br><br>3.48 plants/m^2	• Highest yields were obtained from *Pseudomonas putida* and *Serratia marcescens*<br>• IAA production<br>• *Fusarium* antagonism<br>• Native PGPR strains resulted in higher yield increases than the *Bacillus* inoculant	Gül, 2013 [122]
Cucumber	• *Pseudomonas chlororaphis*<br>• *Trichoderma harzianum* (RootShield Drench)<br>• *Streptomyces griseoviridis* (Mycostop)<br>• *Gliocladium catenulatum (Prestop WP, Prestop Mix)*<br>• *Trichoderma (Gliocladium) virens* (SoilGard)	Decreased mortality > 50%	5 inoculants, 3 media types, 2 fungicides	Up to 40 days	10 × 10 × 7 cm high rock wool blocks in individual plastic bag	• *G. catenulatum* and *Pseudomonas chlororaphis* reduced seedling mortality from *Fusarium* under varying experimental conditions<br>• *G. catenulatum* and *Streptomyces* were more effective than *Trichoderma* at preventing *Pythium* root rot and damping off	Rose, 2003 [137]<br><br>Punja, 2003 [136]
Cucumber	• *Trichoderma harzianum*	~42% increase in dry shoot weight<br><br>No data on fruit	20 plants x 5 containers per treatment<br><br>PGPR+ control Repeated twice	28 days	Autoclavable transparent polycarbonate boxes<br><br>500mL, adjusted daily	• Increased P, Fe, Cu, Mn, Zn after inoculation	Yedidia, 2001 [116]

Duckweed	• *Acinetobacter calcoaceticus*	1.9-2.3x more	20-30 fronds/ 100mL	7 days	1L and 100mL containers<br><br>Repeated batch experiments with re-colonization	• PGPB was involved with phosphorus and nitrogen cycling	Ishizawa, 2020 [100]
Duckweed	• *Aquitalea magnusonii*	11.7-32.1%<br><br>5/9 experiments	10 fronds/60mL<br><br>10^4 and 10^6 inoculation doses<br><br>9 replicates for 12 different groups	7 days	60mL Erlenmeyer flasks	• *Aquitalea magnusonii* significantly increased duckweed growth<br>• PGPB quantities decreased after 7 days	Ishizawa et al., 2020 [104]
Lettuce<br><br>Black Seeded Simpson<br><br>Bibb	• *Gluconacetobacter diazotrophicus*	8.2-16.2%	Two factor fixed effect randomized<br><br>1 PGPB +control<br><br>6 levels fertilizer<br><br>3-4 replicates repeated twice	30-31 days	Kratky hydroponic jars<br><br>44 jars/chamber<br><br>Foil wrapped, 800mL	• *Gluconacetobacter diazotrophicus* increased lettuce yields<br>• IAA, gibberellins	Sebring, 2022 [125]

Lettuce<br><br>Cortina	• Boost (*Bacillus subtilis*)<br>• *Enterobacter aerogenes*<br>• Rootshield (*Trichoderma harzianum*)<br>• Soilguard (*Gliocladium virens*)	Boost increased fresh lettuce weight by 30% in presence of *Pythium*	Random design<br><br>11 treatments<br><br>3 replicates<br><br>3 troughs per treatment	28 days	Recirculating troughs	• *Bacillus subtilis* increased fresh lettuce weight<br>• Mechanism against *Pythium* was unknown, suspected induction of plant resistance genes, general plant growth promotion, or preventing *Pythium* colonization	Utkhede, 2000 [132]
Lettuce<br><br>Crispa	• 1.5 g/L of TNC Bactorr containing 12 *Bacillus* spp. and *Paenibacillus polymyxa*	Prevented ~15% decrease in yields from salt stress	4 treatments or controls<br><br>4 replicate tanks/group<br><br>150 plants/tank	20-22 days	Floating system<br><br>Drilled polystyrene panels<br><br>300 plants/m^2	• Alleviate salt stress (20mM NaCl)<br>• Plants tolerated salinity better in autumn trial compared to spring<br>• Inoculant increased yields	Moncada, 2020 [146]

Lettuce<br><br>Grand Rapids	• Tested shifts in native rhizosphere community over time. Main organisms include:<br>• *Pseudomonas*<br>• *Burkholderia*<br>• *Sphingomonas*	K-struvite had similar yields to traditional NPK fertilizer	Randomized complete block design<br><br>6 plants, 3 replicates<br><br>3 urine-derived fertilizers	29 days	2L pots with rockwool<br><br>12.5 plants/m^2<br><br>Manual irrigation	• K-struvite supported rhizosphere communities and had highest nitrogen levels<br>• ED concentrate and hydrolyzed urine treatments both had distinct microbial communities and were worse treatments for the crop<br>• The most complex microbial community was not always the best one for plant growth	Van Gerrewey, 2021 [97]
Lettuce<br><br>Linda	• *Trichoderma viride*<br>• *Bacillus thuringiensis*<br>• *Pseudomonas fluorescens*	70.3% inhibition of bacterial wilt	7 treatments + control	Unknown	Deepwater culture 30”x18”<br><br>Continuous aeration	• Combining the three PGPB was the most effective combination to reduce bacterial wilt disease caused by *Ralstonia solanacearum*	Khan, 2018 [138]
Lettuce<br><br>Red Cherokee	2.41g commercial Sanolife mix<br>• *Bacillus* spp.	314% increase in growth, however low light and chlorosis were reported in all groups	6 replicate units<br><br>*Bacillus* + control<br><br>3 replicates per treatment	6 weeks	NFT system: 10L<br><br>100L aquaponics tank with tilapia	• Phosphorus solubilization<br>• *Bacillus* influenced nitrogen cycle including ammonia, nitrite, and nitrate levels	Da Silva Cerozi, 2016 [105]

Lettuce<br><br>Red coral<br>Green oak	• *Bacillus velezensis*	~46-244% increase in growth in *Pythium* contaminated lettuce	8x25 plants per treatment<br><br>3 treatments plus non-treated controls	40 days	Dynamic root floating technique 1000L<br><br>60 × 94 cm<br><br>24 platforms/unit	• *Bacillus* reduced *Pythium* root colonization	Kanjanamaneesathian, 2014 [133]
Lettuce<br><br>Romaine	• *Pseudomonas chlororaphis*	17% in weight	8 treatments<br><br>24 replicates per treatment	10 weeks	Window farm/ drip system	• Changed levels of cellular and metabolic gene expression in lettuce<br>• Involved in stress reduction and *Pythium* prevention	Lee, 2015 [16]
Lettuce<br><br>Tiberius romaine	• *Azotobacter chroococcum*<br>• *Azospirillum brasilense*<br>• *Pseudomonas fluorescens*<br>• *Bacillus subtilis*<br>• *Aspergillus niger*	~7-21% increases in total fresh weight	Nested design<br><br>50%, 75%, 100% nutrient solution<br>Second factor:4 experimental groups per nutrient level	28/42 days after planting<br><br>Transplanted at on day 21	Drip irrigation system<br><br>Average discharge of 3.6 L/h	• Inoculation reduced the density of indigenous rhizopheric bacteria<br>• Inoculant increased leaf thickness, area, and macronutrient uptake<br>• Bacteria+fungus results in larger yield increases than with group alone	Aini, 2019 [106,112]

Maize	• *Trichoderma virens*	Reduced disease severity	2 inoculant strains	14 days	300mL<br><br>Polycarbonate culture boxes (10.9 by 10.9 by 15.7 cm)<br><br>16 seedlings/unit	• T. virens provided protection against the pathogen *Colletotrichum graminicola*<br>• The PGPR produces Sm1, which induces plant defense mechanisms<br>• Jasmonic acid genes were induced	Djonović, 2007 [140]
Pepper<br><br>Cubico (sweet)	• *Pseudomonas chlororaphis*	12-20% increase in fresh shoot mass<br><br>No data on fruit	Predisposed vs non plants<br><br>± *Pseudomonas* ± *Pythium*<br><br>3-4 replicates	Up to 19 days	Single plant units<br><br>475mL<br><br>Aerated via bubbler	• *Pseudomonas* significantly increased growth of peppers with and without *Pythium*<br>• *P. chlororaphis* delayed root browning	Sopher, 2011 [131]
Potato	• *Rhodococcus*	Reduced maceration to 20% of control plants	2 systems, 300 plants	14 weeks	Two systems<br><br>One 13L batch system holding 100 plants<br><br>Larger system with long gutters held 200 plants (PHS)	• Quorum sensing degradation of *Pectobacterium* resulted in biocontrol<br>• Gamma-caprolactone treatment was used to initiate the biocontrol	Cirou, 2011 [139]

Potato (P)<br><br>Soybean (S)<br><br>Durum (D) & bread (B) wheat	Myco Madness: 0.38 g/L<br>● 48 taxa isolated<br>● 7 fungi<br>● 41 bacteria<br>● 85% was the fungi *Mucor*	Measured but not reported; focus was on community structure	Sampled systems every 2 weeks to determine microbial community shifts	103 days (DW)<br><br>120 days (BW)<br><br>114 days (P)<br><br>98 days (S)	NFT systems	● Microbial communities of inoculated plants were more stable over time<br>● The Myco Madness product had unexpected composition compared to label<br>● *Pseudoalteromonas* sp. were the most effective colonizers from the commercial mix<br>● Crop specific trends after receiving the same microbial inoculant	Sheridan, 2017 [99]
Rice	From 305 total strains, 30 were tested further. TY0307 (genus and species not provided) was the best growth promoter	~30%	30 strains screened<br><br>10 plants/flask<br><br>3 replicates	10 days	Flasks containing 150mL	● ACC deaminase<br>● Salt tolerance<br>● TY0307 was the most effective isolate<br>● Increase proline and reduced ROS stress	Zhang, 2018 [120]
Rice	● *Bacillus amyloliquefaciens*	15.4%	1 inoculant + control<br><br>Regular and 200mM salt []<br><br>6-12 replicates	10-30 days	15mL conical tube floating	● PGPB upregulated 14 genes, induced salt tolerance<br>● During salt stress, the microbial community shifted to organisms that use osmoprotectants such as trehalose	Nautiyal, 2013 [147]

Rice	• *Pantoea agglomerans*<br>• *Lelliottia amnigena*<br>• *Enterobacter* sp.	~20%	8 plants<br><br>6 replicates	9 days	1.2L container<br><br>Solution renewed every 3 days	• IAA, nitrogen fixation, phosphorus solubilization<br>• *Pantoea agglomerans* reduced cadmium concentrations and increased yield	Tian, 2022 [113]
Rice<br><br>KDML105	• *Pseudomonas stutzeri*<br>• *Cupriavidus taiwanensis*<br>• *Delftia acid ovorans*	50% arsenic reduction	3 replicates<br><br>5 inoculants	90-135 days	10 × 10 ×15 cm containers containing 300mL	• *Cupriavidus taiwanensis* and *Pseudomonas stutzeri* was the optimal combination to reduce arsenic toxicity in rice<br>• Root sulfide accumulation increased to transition arsenic into a harmless arsenic sulfide form<br>• IAA, phosphorus solubilization, nitrogen fixation, ACC deaminase	Thongnok, 2021 [126]

Sedum alfredii	• *Pseudomonas fluorescens*	Increased lateral root formation	1 inoculant + control<br><br>4 replicates	4 weeks	1L container	• *Pseudomonas fluorescens* promoted cadmium uptake<br>• 146 plant hormone genes were upregulated<br>• IAA, ACC deaminase<br>• Decreased multiple compounds including jasmonic and abscisic acid	Wu, 2020 [128]
Sorghum	12 species total tested:<br>• *Pseudomonas* spp.<br>• *Bacillus* spp.<br>• *Burkholderia* spp.<br>• *Phyllobacterium*<br>• *Chitinophaga japonensis*	7-140%	12 inoculants + control<br><br>6 replicates	4 weeks	Tyndallized vermiculite<br><br>Watered every other day	• IAA, siderophores, phosphorus solubilization<br>• IAA producing *Pseudomonas* strains resulted in the greatest increase in plant yields	Amora-Lazcano, 2022 [108]
Soybean	Myco Madness mix<br>• 14 bacteria<br>• Yeasts<br>• 12 fungi	29.9% increase in plant growth<br><br>36.9% increase in seed yield	48 per group<br><br>16/double gully x 3 rep	98 days	NFT system<br><br>Double gully<br><br>Polyprop. 21L reservoir	• Higher density stomata<br>• Thicker palisade parenchyma<br>• Better maximal PSII photochemical efficiency	Paradiso, 2017 [114]

Strawberry<br><br>Pájaro	• *Gluconacetobacter diazotrophicus*<br>• *Azospirillum brasilense*	Reduced iron + inoculant was best for plant nutrition and phenolic compounds	3x3 factorial randomized design<br><br>2 inoculants + control<br><br>3 iron levels<br><br>10 replicates	60 days	100mL glass container covered with aluminum foil	• Siderophore production<br>• Hydroxamate siderophores were more beneficial than catechol siderophores	Delaporte-Quintana, 2020 [118]
Switchgrass	• *Pseudomonas grimontii*<br>• *Pantoea vagans*<br>• *Pseudomonas veronii*<br>• *Pseudomonas fluorescens*	22-112% fresh shoot increase	6 replicate pots<br><br>7 plants per pot<br><br>4 PBPR plus a consortium	Up to 45 days	1.5 L polypropylene pots	• PBPB inoculated plants had elevated HSP70 and HMA3 genes under cadmium stress (20 µmol/L)<br>• IAA production, ACC deaminase, phosphorus solubilization<br>• *Pseudomonas fluorescens* and *Pseudomonas veronii* resulted in the largest increase in fresh shoot weight	Begum, 2018 [109]<br><br>Begum, 2019 [110]
Tomato<br><br>Bandita<br><br>Kardelen	• *Pseudomonas fluorescens*<br>• *Pseudomonas putida*	~10-26% fruit mass increase	Randomized complete block design<br><br>2 cultivars, 4 PGPR+1 control<br><br>12 plants/group	6 weeks	Drip irrigation system<br>2L/h flow rate	• IAA, phosphorus solubilization, siderophores, HCN<br>• *Pseudomonas* strains significantly increased tomato plant and fruit yields	Gül, 2012 [111]

Tomato<br><br>Belladonna F1 grated onto self and M82	• *Paenibacillus*<br>• *Enterobacter mori*<br>• *Lelliottia sp.*	No impact on fruit biomass	Randomized block design with three factors<br><br>4 PGPB<br><br>3 replicates	55 days post transplant	Open hydroponic system in pots<br><br>Perlite growing medium	• *Enterobacter mori* had the highest plant vegetative biomass under stressed and control conditions<br>• Trehalose biosynthesis was increased by PGPB application	Kalozoumis, 2021 [148]
Tomato<br><br>Cherry<br><br>Golden Gem	PGPR:<br>• *Azotobacter chroococcum*<br>• *Azospirillum brasilense*<br>• *Pseudomonas fluorescens*<br>• *Bacillus subtilis*<br>• *Aspergillus niger*<br><br>AMF<br>• *Glomus*	PGPR=29%<br><br>AMF=64%<br><br>Both=48%	Completely randomized nested design<br><br>3 nutrient []s<br><br>3 inoculants and control	Unknown	Drip irrigation 3.6L/h<br><br>Transplanted to polybags containing sand, rice straw charcoal, and compost on day 28	• Fungal applications resulted in the highest fruit weight and diameter compared to bacteria or fungi + bacteria<br>• Higher fertilizer increased sugar content and reduced total titrated acid: phosphate availability is involved in this process. Inoculants increased sugar content and titrated acid.	Aini, 2019 [106,112]

Tomato<br><br>Ingvar	• *Pseudomonas fluorescens*<br>Three commercial mixes:<br>• Binab T (*Trichoderma polysporum* and *T. harzianum*)<br>• Gliomix (*Gliocladium cantenulatum*)<br>• Mycostop (*Streptomyces griseoviridis*)	>50% decrease in disease incidence<br><br>~30-50% increase in dry shoot weight	6 treatments: 4 inoculants, pathogen exposure only, no pathogen<br><br>4 replicates	4 weeks	Static aerated culture system<br><br>1L containers	• All tested products were effective against *Pythium*<br>• Binab T and Mycostop were effective against *Fusarium*	Khalil, 2010 [134]
Tomato<br>Saturn<br><br>Ponderosa<br><br>Lettuce<br><br>Saradana	• Unspecified soil-derived ammonifers and nitrifiers	No significant difference	3 replicates per treatment<br><br>4 plants per pot<br><br>Inorganic vs organic vs fish based fertilizers	Various measurements recorded up to 70 days	1L hydroponic pots<br><br>Aerated with pumps	• Soil based microorganisms that were able to convert organic nitrogen to nitrate were cultured<br>• Outlined a method to use organic fertilizer in hydroponic instead of inorganic	Shinohara, 2011 [107]
Tomato<br><br>Vision	• *Pseudomonas fluorescens* strains	5.6-9.6% in spring<br><br>13.3-18.2% in fall	Randomized complete block<br><br>3 plants per grow bag<br><br>5 replicates	Fruit collected 1-2x per week	24L peat mix growbags	• Fall crop has significantly larger crop yield increases than the spring<br>• *P. fluorescens* strain 63-28 was the most beneficial<br>• Unknown mechanism: no effect on shoots at transplant stage	Gagné, 1993 [127]

Wheat	Cyanobacteria:<br>• *Calothrix* sp.<br>• *Anabaena cylindrica*<br>PGPB:<br>• *Chryseobacterium balustinum*<br>• *Pseudomonas simiae*<br>• *Pseudomonas fluorescens*	36-80%	13 treatments<br><br>3 controls<br><br>5 replicates per group	17 days	Truncated plastic Eppendorf microtube (seedings)<br><br>Day 2: 50mL test tubes	• IAA production<br>• The consortium of five organisms produced the greatest increase in growth	Kholssi, 2021 [123]
Wheat	18 tested in total including:<br>• *Bacillus* sp.<br>• *Halobacillus* sp.<br>• *Staphylococcus succinus*<br>• *Zhihengliuella halotolerans*<br>• *Oceanobacillus oncorhynchi*<br>• *Exiguobacterium aurantiacum*<br>• *Halomonas* sp.<br>• *Virgibacillus picturae*<br>• *Thalassobacillus* sp	Reduced growth reduction from salt stress from 58.4% to 15.6%.	Control + 18 strains<br><br>4 salt []s<br><br>Replicated 3 times	Day 10 after germination	Unspecified<br><br>Aerated with pumps	• Alleviate salt stress (200mM NaCl)<br>• IAA, ammonia production, nitrogen fixation, phosphorus solubilization, ACC deaminase<br>• The most effective organisms were *Thalassobacillus* sp. *Bacillus* sp., *Halomonas* sp., *Oceanobacillus* sp., *Zhihengliuella* sp.and *S. succinus*	Orhan, 2016 [121]

Note: Although the review predominantly focuses on plant growth promoting bacteria, key studies that tested both fungi and bacteria were included to provide a thorough update on the current state of hydroponic research.
